# Prolonged SARS-CoV-2 Positivity in Immunocompetent Patients: Virus Isolation, Genomic Integrity, and Transmission Risk

**DOI:** 10.1128/Spectrum.00855-21

**Published:** 2021-11-17

**Authors:** Isabela de Carvalho Leitão, Pedro Telles Calil, Rafael Mello Galliez, Filipe Romero Rebello Moreira, Diana Mariani, Anna Carla Pinto Castiñeiras, Gustavo Peixoto Duarte da Silva, Richard Araújo Maia, Isadora Alonso Corrêa, Fábio Luís Lima Monteiro, Marcos Romário Matos de Souza, Cássia Cristina Alves Gonçalves, Luiza Mendonça Higa, Liane de Jesus Ribeiro, Vinicius Wakoff Pereira Fonseca, Victoria Cortes Bastos, Carolina Moreira Voloch, Débora Souza Faffe, Orlando da Costa Ferreira, Amilcar Tanuri, Terezinha Marta Pereira Pinto Castiñeiras, Luciana Jesus da Costa

**Affiliations:** a Laboratório de Genética e Imunologia das Infecções Virais, Departamento de Virologia, Instituto de Microbiologia Paulo de Góes, Universidade Federal do Rio de Janeiro, Rio de Janeiro, Brasil; b Departamento de Doenças Infecciosas e Parasitárias, Faculdade de Medicina, Universidade Federal do Rio de Janeiro, Rio de Janeiro, Brasil; c Laboratório de Virologia Molecular, Instituto de Biologia, Universidade Federal do Rio de Janeiro, Rio de Janeiro, Brasil; d Instituto de Biofísica Carlos Chagas Filho, Universidade Federal do Rio de Janeiro, Rio de Janeiro, Brasil; e Hospital Universitário Clementino Fraga Filho, Universidade Federal do Rio de Janeiro, Rio de Janeiro, Brasil; University of Georgia

**Keywords:** COVID-19, SARS-CoV-2, COVID-19 nucleic acid testing, virus shedding, immunocompetent, persistence

## Abstract

Current guidelines for patient isolation in COVID-19 cases recommend a symptom-based approach, averting the use of control real-time reverse transcription PCR (rRT-PCR) testing. However, we hypothesized that patients with persistently positive results by RT-PCR for severe acute respiratory syndrome coronavirus 2 (SARS-CoV-2) could be potentially infectious for a prolonged time, even if immunocompetent and asymptomatic, which would demand a longer social isolation period than presently recommended. To test this hypothesis, 72 samples from 51 mildly symptomatic immunocompetent patients with long-lasting positive rRT-PCR results for SARS-CoV-2 were tested for their infectiousness in cell culture. The serological response of samples from those patients and virus genomic integrity were also analyzed. Infectious viruses were successfully isolated from 34.38% (22/64) of nasopharynx samples obtained 14 days or longer after symptom onset. Indeed, we observed successful virus isolation up to 128 days. Complete SARS-COV-2 genome integrity was demonstrated, suggesting the presence of replication-competent viruses. No correlation was found between the isolation of infectious viruses and rRT-PCR cycle threshold values or the humoral immune response. These findings call attention to the need to review current isolation guidelines, particularly in scenarios involving high-risk individuals.

**IMPORTANCE** In this study, we evaluated mildly symptomatic immunocompetent patients with long-lasting positive rRT-PCR results for SARS-CoV-2. Infectious viruses were successfully isolated in cell cultures from nasopharynx samples obtained 14 days or longer after symptom onset. Indeed, we observed successful virus isolation for up to 128 days. Moreover, SARS-CoV-2 genome integrity was demonstrated by sequencing, suggesting the presence of replication-competent viruses. These data point out the risk of continuous SARS-CoV-2 transmission from patients with prolonged detection of SARS-CoV-2 in the upper respiratory tract, which has important implications for current precaution guidelines, particularly in settings where vulnerable individuals may be exposed (e.g., nursing homes and hospitals).

## INTRODUCTION

The ongoing COVID-19 pandemic has accumulated a total of 234,809,103 cases and 4,800,375 deaths as of 5 October 2021 (1). Brazilian cases represent nearly 10% (21,459,117) of worldwide cases with a case fatality rate of 2.79% (1). The scenario is particularly critical in the state Rio de Janeiro, where this rate reaches 5.15% (2) and less than 45% of the population is fully vaccinated (3). In the absence of a broadly effective treatment and considering the limited availability of vaccines and a protection rate varying from 50 to 92% for the vaccines in use (4), nonpharmacological measures and social isolation of infected individuals are still very important strategies for disease control.

The viral load in the upper respiratory tract (URT) of infected individuals peaks from 1 to 2 days before, to 5 days after symptom onset (DASO), decreasing over the next 1 to 3 weeks (5–7). Despite the detection of viral RNA by real-time reverse transcription PCR (rRT-PCR), most studies failed to demonstrate the presence of replication-competent viruses from URT samples obtained from longer than 8 to 9 days after symptom onset (8, 9), with exceptions described in immunosuppressed and severe cases (10–14). Following these findings, current guidelines recommend discontinuation of isolation after 10 days of symptom onset, without testing (15, 16). However, the presence of intact full-length severe acute respiratory syndrome coronavirus 2 (SARS-CoV-2) genomes in persistently positive patients has been documented previously (17), suggesting the infectious potential of these viruses (18).

To shed light on the issue, we designed a study on mildly symptomatic immunocompetent patients to establish the frequency of replication-competent viruses in URT samples positive for SARS-CoV-2 RNA collected 14 days or longer after symptom onset. Infectious viruses were isolated successfully from 34.38% (22/64) studied samples, and complete SARS-COV-2 genome integrity was demonstrated. We found that 27.45% (14/51) of patients shed infectious virus in the URT, pointing out the risk of continuous SARS-CoV-2 transmission from patients with prolonged detection of SARS-CoV-2 in the URT, which may have implications for redefining isolation policies.

## RESULTS

### Patients show persistently positive rRT-PCR results in control testing.

**FIG 1:**
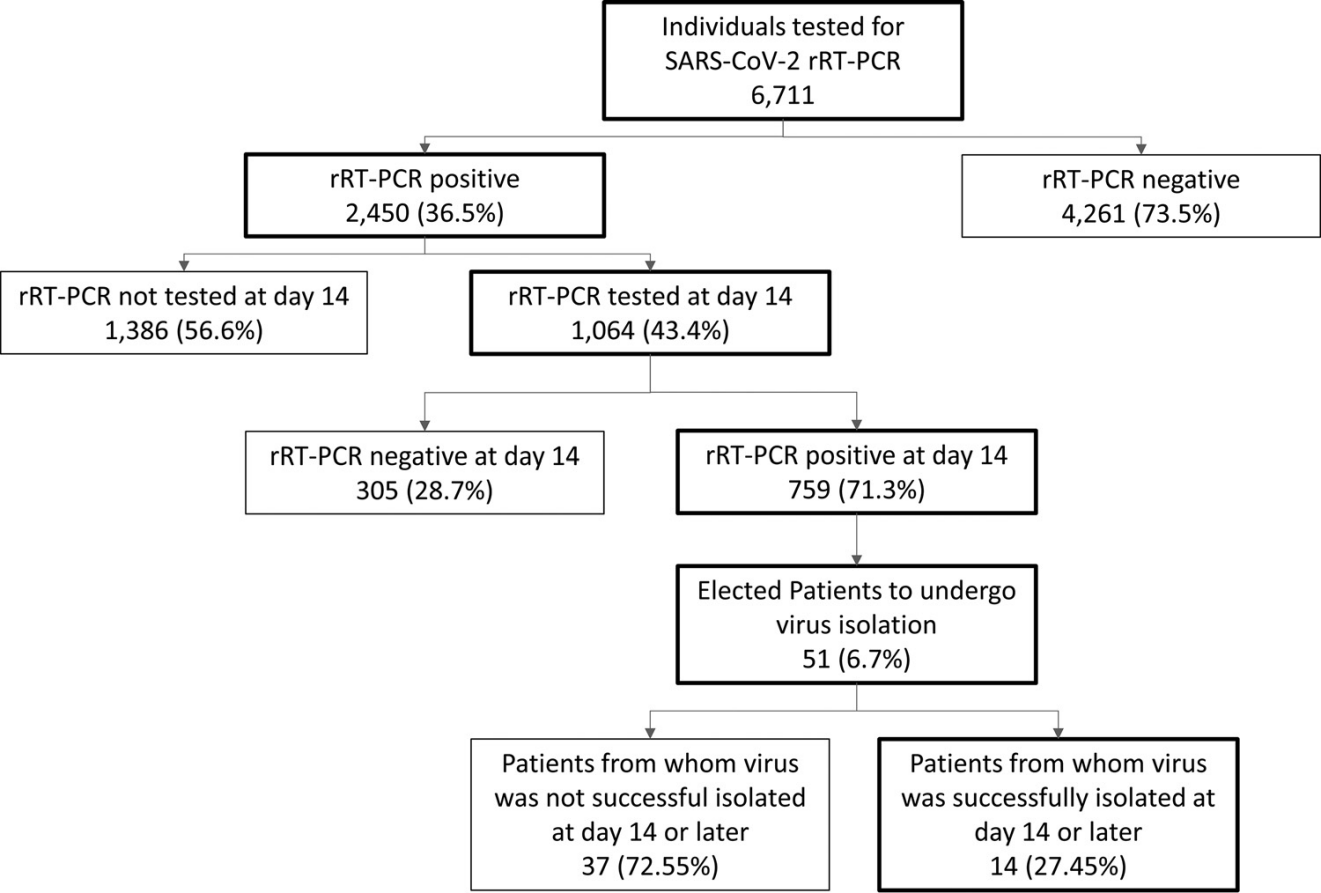
Patient enrollment and sample selection. The figure demonstrates the number of patients that had samples tested by rRT-PCR for SARS-CoV-2 and remained positive after 14 days and the percentage from these samples that were selected for virus isolation in cell culture.

**FIG 2:**
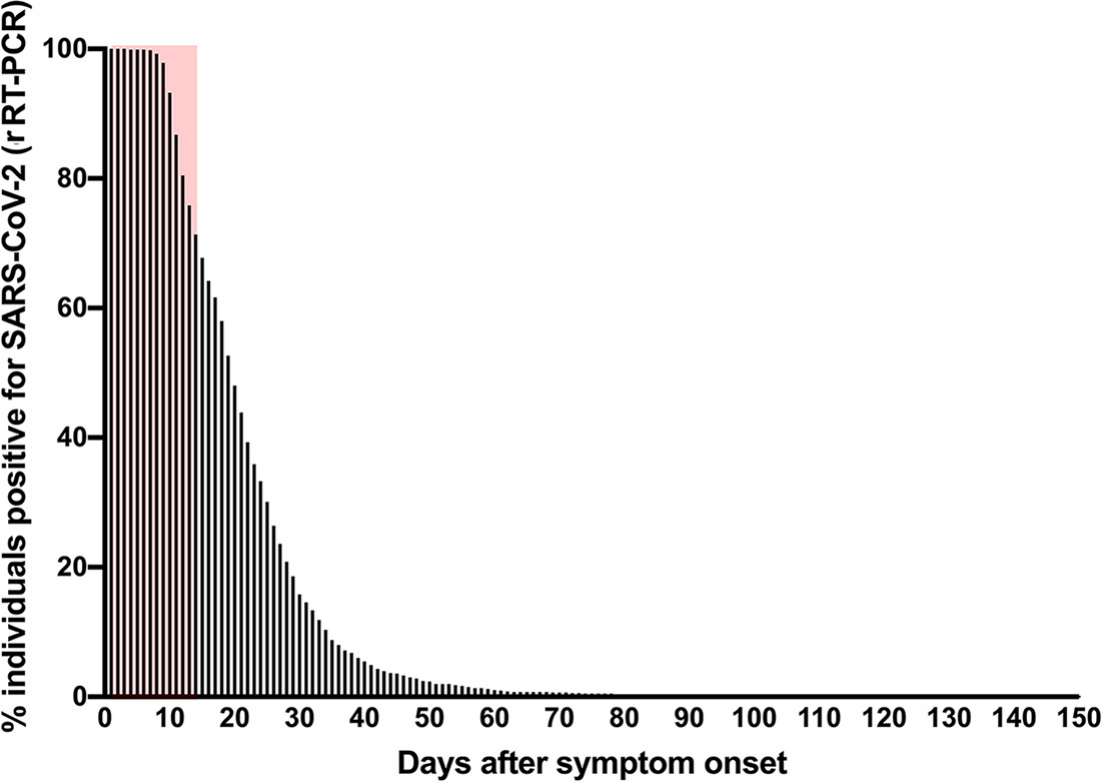
Percentage of patients with persistently positive SARS-CoV-2 rRT-PCR result by days after symptom onset (DASO). Results are the percentage of COVID-19 patients (*n* = 1,064) followed until a negative rRT-PCR result was achieved. Red area represents the percentage of patients with persistently positive rRT-PCR until 14 DASO (71.33%). The longest documented positive rRT-PCR persistence was at 144 days after symptom onset (one patient).

From 18 March to 18 August 2020, 6,711 individuals were tested for SARS-CoV-2. A total of 2,450 (36.50%) individuals tested positive, of which 43.42% (1,064/2,450) were followed until a negative rRT-PCR result was achieved, and then they were further analyzed for positive rRT-PCR persistence (Fig. 1). For the purpose of this study, the rRT-PCR-positive period was determined as the date between the last positive and first negative rRT-PCR result. On the 14th, 21st, and 30th day after symptom onset, 71.33% (759/1,064), 43.89% (467/1,064), and 15.78% (168/1,064) of patients, respectively, had positive rRT-PCR results. The longest rRT-PCR-positive period was 144 days, and the median was 19 days (Fig. 2).

Among the 1,064 persistently positive individuals, 783 (73.45%) had a clinical follow up; 12 (1.53%) individuals required hospitalization in a clinical ward, 10 (1.27%) were admitted in the intensive care unit (ICU), and 3 (0.38%) required mechanical ventilation. No COVID-19-related deaths were reported.

We selected nasopharyngeal (NP) swab specimens only from patients from which none of the samples had been previously unfrozen. Samples from 51 patients with persistently positive rRT-PCR results underwent viral cell culture (Fig. 1). In this group, 60.78% of patients were women, with a mean age of 42.18 ± 12.9 years old, and 47.06% had no comorbidities. Arterial hypertension was the most prevalent comorbidity (23.53%), while no immunosuppressive disease/condition was reported. All patients presented only mild symptoms at their first visit. Fifty-seven percentage of patients were asymptomatic and 37% oligosymptomatic at their first control visit (Table 1). No patient required hospitalization (data not shown).

**TABLE 1:** Population clinical characteristics^*a*^

Characteristic	Viral isolation results (*n*[%])	
	Positive	Negative
Sex		
Female	9/14 (64.29)	22/37 (59.46)
Male	5/14 (35.71)	15/37 (40.54)
Age (yrs)		
Mean ± SD	42.28 ± 11.61	40.72 ± 9.97
<50	12/14 (85.71)	28/37 (75.68)
>50	2/14 (14.29)	9/37 (24.32)
Comorbidities		
None	8/14 (57.14)	16/37 (43.24)
Arterial hypertension	4/14 (28.57)	8/37 (21.62)
Asthma	2/14 (14.29)	5/37 (13.51)
Diabetes	2/14 (14.29)	1/37 (2.70)
Clinical presentation		
Asymptomatic	9/14 (64.29)	20/37 (54.05)
Oligosymptomatic	5/14 (35.71)	14/37 (37.84)
Symptomatic	0/14 (0)	2/37 (5.41)
No information	0/14 (0)	1/37 (2.70)

a Statistical analyses were performed for direct comparisons of all characteristics (sex, age, comorbidities, and clinical presentation) and differences were not significant.

### Nasopharyngeal tissue harbors SARS-CoV-2 infectious virus for a prolonged time.

**FIG 3:**
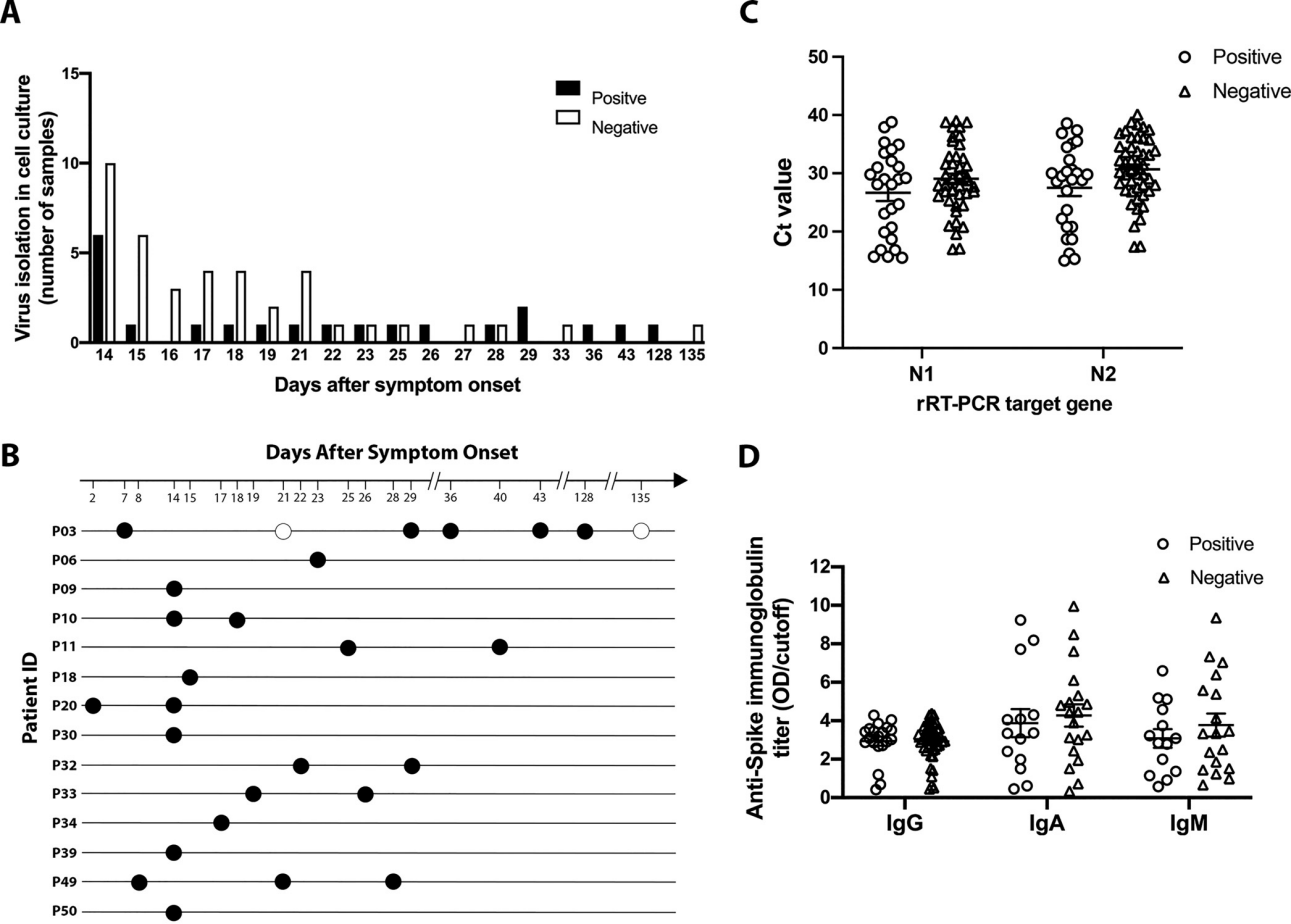
Frequency of viral isolation and humoral response in SARS-CoV-2 PCR+ persistent samples. (A) Number of nasopharyngeal swab samples with positive and negative viral isolation in culture from persistent SARS-CoV-2 PCR+ patients collected at 14 days after symptom onset or longer. Samples (*n* = 61) were inoculated in susceptible Vero E6 and 293/ACE2 cells, and viral production was tested by virus plaque-forming assay. Black bars represent samples with positive viral isolation, and white bars represent samples negative for virus isolation. (B) Distribution of positive (full circle) and negative (hollow circle) viral isolation in culture among NP swab samples from patients with SARS-CoV-2 PCR+ over time (days after symptom onset). Only one sample was available from patients P05, P06, and P10-12. (C) Cycle threshold (*C_T_*) values of rRT-PCR for SARS-CoV-2 N1 and N2 target genes from NP swab samples with positive and negative virus isolation in culture. Lines represent mean ± SEM values. (D) Serum samples time matched with nasopharyngeal swab samples were tested for the presence of IgG, IgA, and IgM against the SARS-CoV-2 spike protein. Dot plots represent optical density (OD)/cutoff values of samples with positive and negative virus isolation. Lines represent mean ± SEM values.

Seventy-two NP samples from the 51 patients, including 8 samples collected before the 14th day after symptom onset, were analyzed. Samples were always subjected to two passages in a blind manner in Vero E6 cells, and virus isolation was confirmed by virus titration of the cell-free supernatant from the second passage. As a confirmation, rRT-PCR was performed on each cell-free supernatant from both the first and the second passage. For those that showed a decrease of at least one cycle threshold (*C_T_*) from the first to the second passage, a third passage in Vero E6 cells was performed and virus isolation was confirmed as before. The 8 samples collected before the 14th day after symptom onset yielded an isolation rate of 62.5%. The 64 samples from 51 patients, collected from the 14th to the 135th day after symptom onset, yielded 34.38% (22/64) and 27.45% (14/51) of positive infectious virus recovery in samples and patients, respectively (Table 2; Fig. 3A; see Table S1 in the supplemental material). Only 2 out of 22 samples positive for virus isolation (9.0%) had virus isolated at the third passage in Vero E6 cells, while infectious viruses were isolated at the second passage from 14 samples (63.6%). When we stratified samples according to the *C_T_* range, the frequency of virus isolation decreased clearly with the increase in *C_T_* (see Fig. S1 in the supplemental material). The rate of infectious viruses recovered from samples with a *C_T_* of >35 was 4.69%, which is equivalent to that shown in the literature (19). The highest recovery was on the 14th day (37.5%) when most samples were available (Fig. 3B). When recovered, infectious viruses were isolated from both Vero E6 and 293T/ACE2 cells. Surprisingly, there was no significant association between rRT-PCR *C_T_* values from samples with negative and positive virus isolation for targets N1 (mean, 26.53 ± 9.98 and 26.67 ± 7.34) or N2 (mean, 30.68 ± 5.49 and 27.51 ± 7.12) (Fig. 3C). A total of 63.6% (14/22) of samples from which infectious SARS-CoV-2 was isolated had *C_T_* values ranging from 15 to 30, while only 9% (2/22) of them had *C_T_* values at 37 to 38 (Fig. S1). From both of these samples, viruses were isolated after 3 passages in Vero E6 cells. For 50.0% (7/14) of patients positive for infectious virus, follow-up samples were analyzed, and infectious viruses were consistently isolated (Fig. 3B). Of note, one patient with a persistently positive rRT-PCR result for 144 days harbored infectious virus in the nasopharynx for up to 128 days after symptom onset (Table 2 and Fig. 3B).

**TABLE 2:** Characteristics of patients persistently positive for SARS-CoV-2 by rRT-PCR with positive virus isolation

Patient-sample	DASO^*a*^	N1/N2 (*C_T_* values)	Virus isolation result	rRT-PCR (N1) (*C_T_* value from cell culture)^*b*^	ELISA-IgG SPIKE (OD/cutoff)^*c*^	Neutralization (EC_50_)
P03-1302	7	18.7/28.6	Pos	16.7	0.42	<1:20
P03-2620	21	29.6/29.8	Neg	38.65	**2.94**	1:100
P03-4117	29	15.7/15.0	Pos	16.7	**3.58**	1:20
P03-5943	36	31.0/30.0	Pos	17.67	**3.39**	1:100
P03-7439	43	28.1/28.9	Pos	16.93	**3.51**	1:20
P03-17056	128	29.8/29.8	Pos	17.33	**4.05**	1:100
P03-17661	135	35.0/36.0	Neg	35.77	**3.59**	1:20
P06-2340	23	16.8/18.7	Pos	23.18	**2.45**	<1:20
P09-3094	14	23.1/23.7	Pos	18.47	**2.14**	1:20
P10-3663	14	31.1/30.6	Pos	14.3	**2.90**	<1:20
P10-4600	18	29.0/30.3	Pos	14.1	ND	ND
P11-3664	25	19.9/20.8	Pos	14.9	**3.47**	ND
P11-6995	40	38.8/38.6	Pos	28.85	**3.65**	<1:20
P18-5191	15	20.7/20.8	Pos	25.58	**2.90**	<1:20
P20-5912	2	15.7/16.2	Pos	19.84	ND	ND
P20-8255	14	34.9/35.1	Pos	15.78	**2.72**	<1:20
P30-8225	14	29.0/29.5	Pos	13.94	**2.86**	<1:20
P32-10311	22	28.1/28.9	Pos	14.72	**3.02**	<1:20
P32-11222	29	24.7/27	Pos	13.76	**3.20**	ND
P33-10406	19	29.2/30.0	Pos	16.89	**2.87**	<1:20
P33-11323	26	34.1/34.5	Pos	18.41	**2.43**	1:100
P34-10417	17	37.9/37.4	Pos	13.98	**2.68**	1:2,500
P39-11507	14	33.5/35.5	Pos	19.03	**3.08**	<1:20
P49-11787	8	15.5/15.3	Pos	14.87	0.68	<1:20
P49-13083	21	32.1/32.3	Pos	15.55	**4.29**	<1:20
P49-13867	28	35.3/36.9	Pos	14.22	**3.86**	1:2,500
P50-12606	14	23.9/22.2	Pos	14.59	1.19	ND

a DASO, days after symptom onset.

b RT-qPCR from cell supernatant after the second passage in culture.

c Results of the inhouse ELISA assay are represented as optical density (OD)/assay cutoff. Bold text, positive values; underlined text, inconclusive values; ND, not determined.

### The serological response did not impact the isolation of infectious virus.

Most patients studied showed a detectable serum anti-spike protein IgG (mean: 3.01 ± 0.93 and 2.88 ± 0.99 in the negative and positive groups, respectively) at 2 weeks after symptom onset (Fig. 3D; Table 2; see Table S2 in the supplemental material). Anti-S IgA and IgM were also readily detected (Fig. 3D). No differences in immunoglobulin profile or relation of the sample optical density value divided by assay cutoff (S/C ratio) between positive and negative virus isolation groups were observed (Fig. 3D). The absence of SARS-CoV-2 neutralizing antibodies was also similar in both groups. The median titer of neutralizing antibodies for all samples was low (<1:20), but both groups had samples with neutralizing titers ranging from 1:100 to 1:2,500 (Table 2 and S2).

### Patients persistently infected with SARS-CoV-2 harbor full-length viral genomic RNA in the nasopharynx.

**FIG 4:**
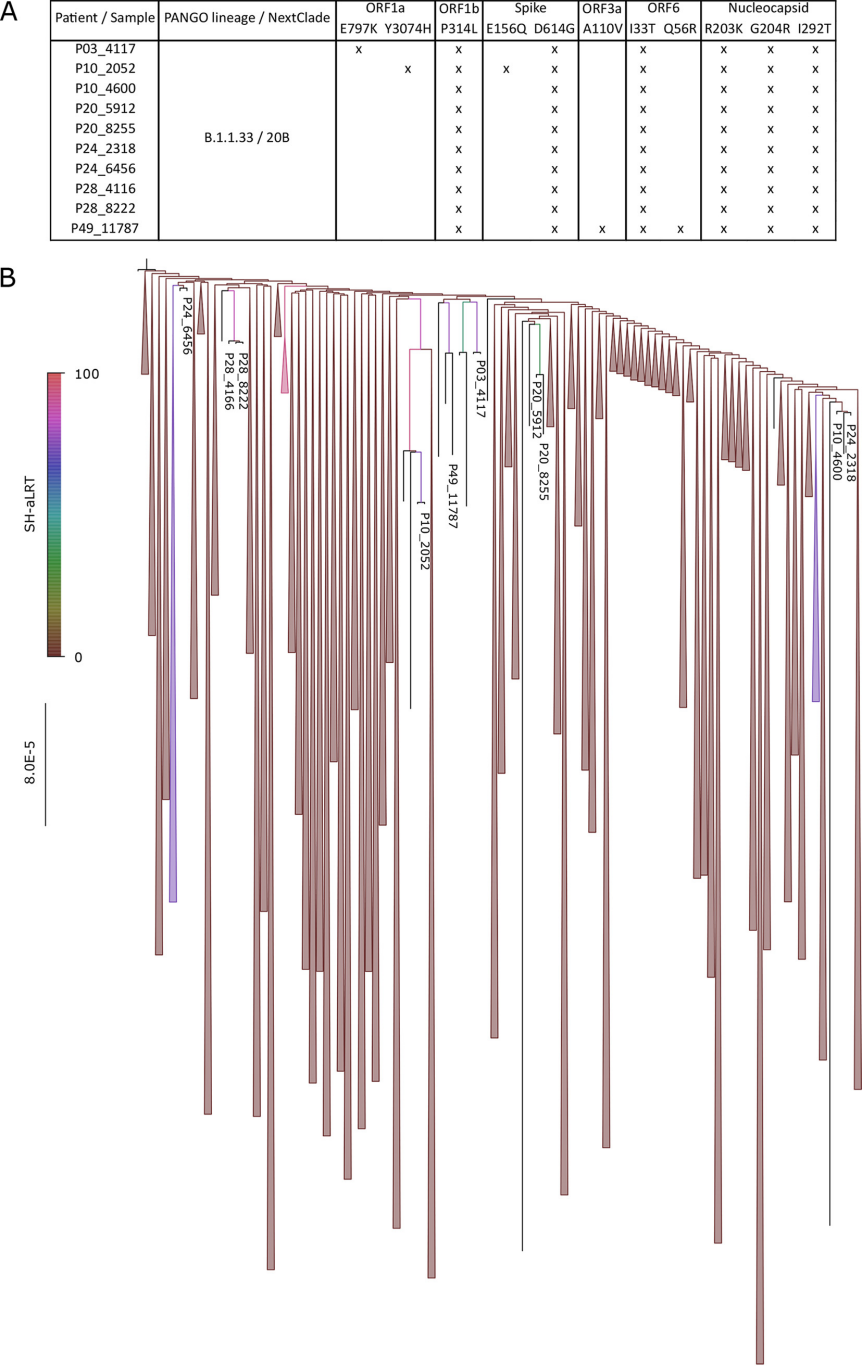
Genomic description and phylogenetic analyses of SARS-CoV-2 from persistently infected immunocompetent patients. (A) Panel exhibiting all nonsynonymous mutations identified in each sequence by Nextclade v1.7.1 (“x” marks presence), indicating the high degree of similarity of the characterized genomes. Noticeably, most pairs of sequences characterized from the same patients did not present any exclusively shared mutation (synapomorphy). (B) Sequences were aligned with a comprehensive set of B.1.1.33 sequences available on the GISAID EpiCoV database (*n *=* *1,538) and a maximum likelihood tree was inferred with IQ-Tree v2.0.3, under the GTR+F+I+G4 model. The tree indicates the novel genome group with diverse branches along the tree, although often with limited support values, likely reflecting the high nucleotide identity in the data set. Only two pairs of samples from patients 20 (5912 and 8255) and 28 (6936 and 8222) cluster together, likely due to the presence of an exclusive synonymous mutation for each, G24040T and A3409G, respectively. Branch colors indicate SH-aLRT support values, as indicated in the color gradient bar on the left. The scale bar marks substitutions per site. Irrelevant branches have been collapsed, and the tree was rooted in the oldest sequence available in the data set for visualization purposes only.

To check for SARS-CoV-2 genome integrity in the NP swab samples, whole-genome sequencing was performed on follow-up samples from patients P03, P10, P20, P24, and P28. For patients P10, P24, and P28, the first samples taken within the first 7 DASO were included in this study only for sequence comparison purposes (samples 2952, 2319, and 4166, respectively); no virus isolations from these samples were performed. The follow-up samples from patients P24 and P28 were negative for virus isolation (Table S1). Unique samples collected 14 days after symptom onset or longer from P03 and P20 were analyzed. The first samples collected within the first 8 DASO were also included and analyzed for both sequence and virus isolation (Table 2). A sequence obtained from the first NP swab sample taken from patient P49 was also included. Genome coverage ranged from 80.15% to 99.80%, while average sequencing depth ranged from 657.07- to 5,885.25-fold. Complete SARS-CoV-2 genomes with intact open reading frames were recovered for all NP swab samples analyzed, suggesting the presence of intact genomic RNA in the persistently infected patients. All 10 sequences were classified as lineage B.1.1.33, which was the prevalent variant in Rio de Janeiro at the time of the study. They exhibit 2 synonymous and 6 nonsynonymous nucleotide mutations characteristic of this lineage (Fig. 4, top panel). A phylogenetic analysis performed with a comprehensive data set of B.1.1.33 sequences revealed the newly characterized genomes clustered with diverse previously characterized genomes, although with limited supported values (Shimoidara-Hasegawa like approximate likelihood ratio test [SH-aLRT] median, 63.85; range, 0 to 91.7) (Fig. 4, bottom panel). This limit on statistical support likely reflects the high similarity among sequences in the data set (*p*-distance, 99.99%). Accordingly, the two follow-up sequences which clustered together with the previous sample from the same patient (P20 and P28) did so because of unique synonymous mutations (G24040T and A3409G, respectively) shared by both (Fig. 4), and this finding supported the persistence of SARS-CoV-2 infection. For patient P24, although its paired sequences were highly homologous, they did not present a synapomorphy. For patient P10, the viral sequence from the first time point had two unique synonymous and two unique nonsynonymous mutations that were not found on the follow-up sample (Fig. 4). These findings suggest prolonged intact virus shedding in the upper respiratory tract of persistently rRT-PCR-positive patients that maintained widespread genomic stability.

Viruses isolated from samples P03-4117, P20-5912, and P49-11787 were also sequenced and compared to the sequences obtained directly from the NP swab sample. Sequences characterized from both sources were identical for each patient (see Fig. S2A and B in the supplemental material), confirming the identity of the viral isolates. Also, plaque characteristics of viral isolates are shown in Fig. S2C. The morphology and size of SARS-CoV-2 plaques were homogeneous among all viral isolates. For instance, no striking difference in viral plaques was noted between viruses isolated earlier and later in the course of the infection for patients P03 and P049 (Fig. S2C). Since viral plaque morphology in cell lines correlates with viral growth capability, these data suggest an equivalent fitness for viruses replicating at distinct times of infection.

The sequence from the virus isolated from the first sample of patient P03 (P03-1302) was compared with the follow-up sequence (P03-4117), and two differences were noted, as follows: (i) unique nonsynonymous substitution G19891 (Orf1b: D2142Y) in P03-1302 and (ii) unique nonsynonymous substitution G2654A (Orf1a: E797K) in P03-4117 (Fig. S2A). Thus, virus recovered from the same patient 22 days apart showed 99.99% homology, confirming the persistence of highly similar viruses in the upper respiratory tract of SARS-CoV-2 persistently infected individuals.

## DISCUSSION

In a setting of high demand for tests, health agency guidelines recommend discontinuation of social isolation 10 days after symptom onset in the absence of fever for at least 24 hours and with symptom improvement, without control testing (15, 16). These recommendations are based on reports of unsuccessful replication-competent virus recovery after 8 to 9 days from symptom onset, suggesting a negligible risk of SARS-CoV-2 transmission (8, 9). The lack of infectious virus isolation has been explained by the presence of fragments of the virus genome and/or defective viral particles (20). In the present study, we showed, by performing viral culture in SARS-CoV-2-susceptible Vero E6 and Hek-AceII cells, that a proportion of NP swab samples that were persistently positive by rRT-PCR for SARS-CoV-2 harbor infectious virus even after 14 days and for as long as 128 days after symptom onset. Moreover, the observation of virus genome integrity supports the presence of intact full-length SARS-CoV-2 genomic RNA.

Few studies have attempted to isolate infectious viruses from prolonged SARS-CoV-2 shedding (8, 19, 21–24). van Kampen and colleagues reported low (5%) infectious virus recovery 15 days after symptom onset or longer (24). Nevertheless, the recovery of cultivable virus up to 18 and 24 days after symptom onset has been reported (14, 23). In the present study, we achieved infectious virus recovery in 27.45% of patients after the 14th day after symptoms onset, including 6 patients with persistent SARS-CoV-2 detection by rRT-PCR for longer than 21 days. This apparent discrepancy could be related to variations in the methodology for virus isolation and sample characteristics. We used two to three consecutive passages in Vero-E6 and 293T/ACE2 cells, which increased our recovery rate by 25%. The rate of virus isolation from acute samples with this methodology in our laboratory varies from 60% to 75% (data not shown), which is higher than the general success ratio reported (24, 25).

Successful virus isolation has been demonstrated mainly in samples with *C_T_* values lower than 25 (8, 11, 19, 24). However, we successfully isolated infectious viruses from samples with a low estimated viral load (*C_T_* value, >32). These data are supported by reports of infectious virus recovery from samples with *C_T_* values of 32 (14, 23) and the previously determined *C_T_* value cutoff of 37 for virus isolation from URT specimens (23). This apparent discrepancy could be due to the differences in viral culture assays implemented which will differ in sensitivity. In our case, two to three consecutive passages in Vero E6 cells were used for infectious virus isolation. In any case, it has been shown previously, in a data set of mildly symptomatic patients, that the probability of virus recovery from samples with *C_T_* of >35 is 8.3% (19), which was similar in our data set. Moreover, the variance in *C_T_* results when using different targets for rRT-PCR (18, 26, 27) and the sampling method quality (28) could also account for this discrepancy. It is noteworthy that a substantial number of our samples collected after 14 days from symptom onset or longer showed *C_T_* values under 28 (55%) and 20 (30%). These *C_T_* values correspond to estimated viral loads of around 10^6^ and 10^10^ copies per mL (29). Previous studies analyzing the temporal dynamics of viral shedding and transmissibility of SARS-CoV-2 report similar *C_T_* values in patients harboring infectious virus and those in viral supercarriers/super spreaders, supporting the increased risk of transmissibility (29, 30).

The use of subgenomic RNA (sgRNA) as a surrogate marker of active viral replication has been proposed (22). However, no correlation between sgRNA detection and cell culture isolation results is unequivocally established (24, 31). This may be due to a prolonged half-life of these RNAs even after viral replication stops. Therefore, it is still not known if individuals who remain sgRNA positive after symptom resolution and seroconversion remain infectious to others. In contrast, although indirectly, the demonstration of virus genomic integrity by sequencing may correlate with the presence of replication-competent viruses and the potential risk of transmission (18). Indeed, our study demonstrated both successful virus isolation and genomic integrity from persistently positive rRT-PCR NP swab samples, reinforcing the risk of continuous SARS-CoV-2 transmission.

Additional arguments supporting the negligible risk of SARS-CoV-2 transmission from 8 to 9 days after symptom onset rely on the fact that most patients diagnosed with COVID-19 present a specific anti-SARS-CoV-2 humoral response, especially neutralizing antibodies against the receptor-binding domain of the spike protein, yielding postinfection immunity (24, 32–34). Nevertheless, viral shedding may persist despite seroconversion (21, 35), which correlates with the findings described here of neutralizing antibodies and specific IgG, IgA, and IgM. Among patients harboring infectious viruses in the NP swab, 36.36% presented neutralizing antibodies and 100% (92.85% positive and 7.14% borderline) had IgG against the SARS-CoV-2 spike protein. Thus, our data indicate clearly the presence of infectious virus on the site of virus shedding, even in the presence of neutralizing antibodies in circulation, highlighting the possibility of continuous viral transmission and underling that serological testing is not appropriate for deciding the adequate end time of isolation.

Evidence from a small case series demonstrates that severe cases can be susceptible to prolonged infections and virus shedding (36, 37), including immunosuppressed pediatric and adult patients, and can be associated with an accelerated viral evolution (38, 39). We demonstrated prolonged persistence of infectious SARS-CoV-2 in immunocompetent individuals with mild disease that were mainly asymptomatic or oligosymptomatic at follow-up testing. Also, to our knowledge, we described the longest-lasting detection (135 days) of rRT-PCR results positive for SARS-CoV-2, as well as the most extensive report of replication-competent virus (128 days) from NP swab samples in immunocompetent individuals. However, despite prolonged virus shedding, we did not observe an expressive accumulation of SARS-CoV-2 mutations in follow-up samples collected up to 22 days apart. Similarly, Li et al. (40) demonstrated the recovery of infectious virus in two persistently positive patients (samples from 73 and 102 days after symptom onset) and few genomic variations from viruses obtained 20 days apart (40).

In our cohort, we showed that 71.33% of the patients persisted with SARS-CoV-2-positive rRT-PCR results by 14 days after symptom onset, lowering substantially by 21 (43.89%) and 30 (15.78%) days. Assuming a viral isolation rate of 27.45% (14/51 cases reported here) and back calculating to the actual 71.33% of the cases with persistently positive PCR by 14 days after symptom onset, we can expect at least 19.58% of all positive individuals to be continuous carriers of infectious virus at 14 days after symptom onset. These results urge for an extended time of isolation and the reinforcement of respiratory precautions, especially in settings where vulnerable individuals may be exposed.

Nevertheless, limitations to our findings need to be considered. First, due to the flexible time interval between sample collections, it was not possible to determine the precise time of virus clearance in each patient. Also, in this study, we did not investigate transmission events from persistently infected individuals to their contacts. Further studies with temporally structured sampling strategies and that explore through sequencing the occurrence of transmission clusters are necessary to bridge these gaps.

In conclusion, our findings support that SARS-CoV-2-infected immunocompetent individuals can shed infectious virus for a prolonged time, regardless of symptoms, comorbidities, NP swab viral load, or humoral response. Accordingly, these data suggest the need to revise current guidelines for COVID-19 precautions, particularly in high-risk settings.

## MATERIALS AND METHODS

### Cohort inclusion criteria.

The Center for COVID-19 Diagnosis was founded at the Federal University of Rio de Janeiro and has been offering diagnostic testing for mildly symptomatic public health care or security force workers in Rio de Janeiro, Brazil. From March to August 2020, we enrolled individuals suspected of SARS-CoV-2 infection. A questionnaire was conducted including demographic, symptom, comorbidity, and exposure risk information. A subset of this cohort, represented by all positive patients, were invited for a control rRT-PCR test 14 days after symptom onset and, if still positive, for a follow-up every 7 days until a negative result was achieved.

Fifty-one patients were selected from those with persistent viral RNA shedding, defined as SARS-CoV-2-positive NP swab samples by rRT-PCR for ≥14 days after symptom onset. This criterion was guided by previous recommendations for the COVID-19 patient isolation period (14 days) (15, 41). From these 51 patients, 72 samples were selected for culture, including 8 samples collected before and 64 at or after the 14th day after symptom onset. The patient and sample selection were based on the persistently positive rRT-PCR results and on sample quality criteria, as follows: (i) all samples from the same patient should have been kept at −80°C since the time of collection and must not have been previously unfrozen, (ii) the availability of a minimum of a 1-mL volume of each sample, and (iii) recovery of at least 50% of the sample volume after filtration through a 0.22 μM Merck Millipore filter. Time resolution of virus shedding was defined as the medium day between the last positive and the first negative rRT-PCR result for each individual.

The present study was approved by the National Committee of Research Ethics (CAAE-30161620.0.1001.5257). All enrolled participants were over 18 years old and declared written informed consent.

### Sample collection.

For all suspected COVID-19 cases, SARS-CoV-2 rRT-PCR and a serological evaluation were performed on nasopharyngeal (NP) samples. NP samples were collected from both nostrils using two rayon-tipped swabs. Material was stored in Dulbecco’s modified Eagle’s media (DMEM; ThermoFisher Scientific) at 4°C until transportation to the laboratory and then stored at −80°C. Time between time collection and sample storage was 2 hours on average.

### RNA extraction and rRT-PCR.

Total viral RNA was extracted using the Maxwell 16 viral total nucleic purification kit System (Promega) according to manufacturer’s instructions. Viral RNA was detected using the SARS-CoV-2 (2019-nCoV) CDC qPCR probe assay (Integrated DNA Technologies, IA, USA) (42) targeting the SARS-CoV-2 N1 and N2 genes and the human RNase P (RNaseP) gene. All reactions were paired and performed in a 7500 thermal cycler (Applied Biosystems, CA, USA). A SARS-CoV-2 rRT-PCR result was considered positive if both targets (N1 and N2) were amplified with a cycle threshold (*C_T_*) of ≤40, inconclusive if only one target was amplified with a *C_T_* of ≤40, and negative if both targets were not amplified or amplified with a *C_T_* of >40.

### Cell culture.

African green monkey kidney cells (Vero E6; ATCC CRL-1586) and human embryonic kidney cells (HEK293T; ATCC CRL-3216) were cultured in DMEM with 10% fetal bovine serum (FBS; Gibco), 100 U/mL penicillin, and 100 μg/mL of streptomycin (ThermoFisher Scientific). HEK293-AceII (293T/ACE2) was a gift from Bieniasz (The Rockefeller University, NY, USA) (43) and was cultured as above. All cell lines were maintained at 37°C with 5% CO_2_ and passed every 3 to 4 days.

### Virus isolation and plaque-forming unit assay.

SARS-CoV-2 was isolated from NP swab samples. Briefly, Vero E6 and 293T/ACE2 cells were plated in 6-well plates to achieve 70% of confluence overnight. Cells were infected with 250 μL (1/4 of the NP sample original volume) of filtered samples diluted 1:2 in DMEM for 1 h to allow virus adsorption. Then, the inoculum was replaced with 5% FBS-supplemented DMEM. Cells were incubated at 37°C and 5% CO_2_ for 72 h (passage 1). Passage 1 culture supernatants were collected from each infection, filtered through a 0.22 μM Merck Millipore filter, and inoculated into fresh cell cultures as described above and incubated for 72 h. Supernatants were collected, filtered, and stored at −80°C for the later detection of SARS-CoV-2 by rRT-PCR. For some samples, a third passage in Vero E6 293T/ACE2 cells was conducted, as described above. Positive viral cultures were defined by the visualization of viral plates after viral titration by plaque forming assay in Vero E6 cells. For virus titration, 10-fold dilutions of each sample stock were used to infect Vero E6 cells in α-MEM supplemented with 1% of fetal calf serum (FCS) and with 1.4% carboxymethylcellulose or 1% agarose for 4 days at 37°C with 5% CO_2_. Then, cells were fixed with 4% formaldehyde and stained with 1% crystal violet in 20% methanol for plaque visualization and quantification.

All cell-free supernatants from the first and second passage were also subjected to viral RNA extraction, and rRT-PCR was performed as described previously for virus isolation confirmation. Samples that were negative for virus isolation after two consecutive passages in each cell line, as defined by the visualization of viral plaque formation but having at least one-*C_T_* decrease from the first to the second passage by rRT-PCR, were subjected to a third passage and considered positive if viral plaques were visualized by the viral plaque-forming assay.

### Pseudovirus neutralization assay.

SARS-CoV-2 pseudovirus production was performed using the lentiviral plasmid for the lentiviral-based pseudotyping system (pNL4-3-ΔEnv-NanoLuc) and the expression vector for the SARS-CoV-2 spike glycoprotein (pSars-CoV-2-S), as described previously (43). Patient serum samples were inactivated at 56°C for 40 minutes and diluted in a 5-fold serial dilution (1:20, 1:100, 1:500, and 1:2,500) in DMEM medium. Infection was detected by the luciferase assay system (Promega, EUA) according to the manufacturer. The serum dilution with 50% effective concentration (EC_50_) was calculated as the percentage of virus replication in each serum dilution divided by the replication of the infected control and plotted as a nonlinear Sigmoidal 4PL curve (Prism 9; version 9.1.0).

### Measurement of anti-SARS-CoV-2 spike protein antibodies in serum.

Microtiter plates (Immulon 2 HB) were coated with trimeric spike proteins (4 μg per mL), incubated overnight at 4°C, and blocked with 5% bovine serum albumin (BSA) in phosphate-buffered saline + 0.05% Tween 20 (PBST, Sigma). Samples diluted at 1/50 in PBST + 2% BSA were incubated (1 hour at 37°C), and after washing, horseradish peroxidase (HRP) conjugated anti-human IgG, IgM, or IgA (Southern Biotech) were added (1 hour at room temperature). The reaction was developed with 3, 3′, 5, 5′-tetramethylbenzidine (TMB; Sigma), stopped with 1 N sulfuric acid, and read at 450 nm (Biochrom Asys reader). Samples from a COVID-19-positive case and from a prepandemic period (in triplicate) were added as controls and for cutoff determination. Results were expressed as a S/C ratio. An S/C ratio of <1 is negative and >1.5 is positive and borderline if between these values.

### Next-generation sequencing and analysis.

Library preparation for sequencing was performed using the TruSeq DNA Nano kit or Nextera DNA Flex kit (Illumina, USA), and viral whole-genome amplification was carried out following the Artic Network protocol (https://artic.network/ncov-2019) (17). Sequencing was performed in a MiSeq system using MiSeq reagent kit v3 (Illumina, USA). The cutoff *C_T_* value for NP swab sample sequencing was 29. Data quality was assessed by FastQC (v0.11.4), low-quality reads (<25) were filtered with trimmomatic v0.39, and sequences were mapped to the reference genome NC_045512.2 using the BWA 0.7.17 software. The consensus genome sequence was generated using bcftools v1.10.2 and bedtools v2. 29.2 packages. Single-nucleotide variants (SNVs) were detected using GATK v4.1.7.0.

The novel genome sequences were classified with the pangolin tool v3.1.11 (pango v1.2.76, pangoLEARN model from 17 September 2021) (44) and the NextClade Web application v1.7.1 (https://clades.nextstrain.org/), which was also used to annotate all mutations. To further contextualize them, a phylogenetic analysis has been performed with maximum likelihood with IQ-Tree v2.0.3 (45), under the GTR+F+I+G4 model (45). A comprehensive data set with all Brazilian sequences from lineage B.1.1.33 from 2020 has been assembled from high-quality data available on the GISAID EpiCoV database (*n* = 1,538; acknowledgment table provided as a supplemental material) (as of 28 September 2021). MAFFT v7.480 (46) was used for multiple sequence alignments, and the Shimoidara-Hasegawa like approximate likelihood ratio test (SH-aLRT) (47) was used to assess branch statistical support. Two analyses have been carried out, with one using only sequences from NP swabs and the other also including sequences characterized from viral isolates.

### Statistical analysis.

Student’s *t* test and chi-square test were used to analyze differences between positive and negative virus isolation groups, using Prism 8.00 (GraphPad Software, Inc., CA, USA). The level of significance was set at 5%.
